# Design and Performance Investigation of a Robot-Assisted Flexible Ureteroscopy System

**DOI:** 10.1155/2021/6911202

**Published:** 2021-11-18

**Authors:** Jianchang Zhao, Jianmin Li, Liang Cui, Chaoyang Shi, Guowu Wei

**Affiliations:** ^1^School of Mechanical Engineering, Tianjin University, Tianjin 300350, China; ^2^Key Laboratory of Mechanism Theory and Equipment Design, Ministry of Education, Tianjin 300350, China; ^3^Urology Department of Civil Aviation General Hospital, Beijing 100123, China; ^4^School of Science, Engineering and Environment, University of Salford, Salford, UK M5 4WT

## Abstract

Flexible ureteroscopy (FURS) has been developed and has become a preferred routine procedure for both diagnosis and treatment of kidney stones and other renal diseases inside the urinary tract. The traditional manual FURS procedure is highly skill-demanding and easily brings about physical fatigue and burnout for surgeons. The improper operational ergonomics and fragile instruments also hinder its further development and patient safety enhancement. A robotic system is presented in this paper to assist the FURS procedure. The system with a master-slave configuration is designed based on the requirement analysis in manual operation. A joint-to-joint mapping strategy and several control strategies are built to realize intuitive and safe operations. Both phantom and animal experiments validate that the robot has significant advantages over manual operations, including the easy-to-use manner, reduced intraoperative time, and improved surgical ergonomics. The proposed robotic system can solve the major drawbacks of manual FURS. The test results demonstrate that the robot has great potential for clinical applications.

## 1. Introduction

**Figure 1:**
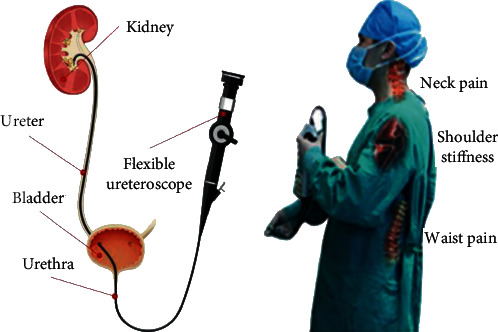
(a) The operational path of FURS in anatomy; (b) the typical physical fatigue and injury caused by the manual FURS manner.

Flexible ureteroscopy (FURS) has been introduced and gradually grown as a routine and effective minimally invasive surgery (MIS) procedure for both diagnosis and therapeutic operations to treat ureteral and kidney stones, strictures, and tumors [1, 2]. Compared with other treatments, such as percutaneous nephrolithotomy (PCNL) and shock wave lithotripsy (SWL), FURS can provide the advantages of a higher stone-free rate, less blood loss, shorter hospitalization, and fewer complications [3–8]. However, the FURS procedure requires strict training practices on both phantoms and animals for professional skills, due to the lengthy and narrow ureters and slender instruments [9–11]. In addition, the long-time standing pose, the heavy load, and the indisposed operation postures during operation lead to causing physical fatigue and burnout for surgeons, lowering treatment quality and decreasing patient safety [12, 13], as illustrated in Figure 1(b). Besides, flexible ureteroscopes and the associated surgical tools are typically expensive surgical instruments with high maintenance costs [14]. Therefore, it is usually operated by surgeons who received special and sufficient training.

To tackle these challenges, the robot-assisted natural orifice transluminal endoscopic surgery (NOTES) techniques have been extensively studied to perform both diagnostic and therapeutic procedures through natural orifices to further reduce incisions and trauma and shorten the learning curve in surgical practice and skill training with accurate and intuitive operation [15–23]. However, compared with other types of robot-assisted NOTES, FURS is featured by the lengthy and thin ureters in anatomy (with a diameter range of 2-5 mm), slender ureteroscopes (with a typical diameter of 3 mm), and fragile operational instruments (with a diameter of around 1 mm) with only 1 or 2 degrees of freedom (DoFs). These features result in complex manipulation and apparent inconsistency between the proximal and distal motions, making it more challenging for robotic control. Therefore, quite a few robotic systems have been designed to target FURS. Among them, the American urologist Desai took the lead in applying the Sensei robotic catheter system (Hansen Medical, CA, USA), which was initially developed for endovascular surgery, to implement robot-assisted FURS and perform renal calculi treatment in 2008 [24, 25]. This robotic catheter system was modified and equipped with a fiber endoscope and a customized ureteral catheter, to perform ureteroscopy with the commercial master device Omega 3 (Force Dimension, Switzerland). However, the existing disadvantages of improper control mode and insufficient workspace partially contributed to the termination and failure of this project [26]. Another robotic system, Roboflex Avicenna (ELMED Inc., Ankara, Turkey), has been specially designed with a commercial flexible ureteroscope to address the clinical demands for FURS in 2014 [27–29]. Clinical trials have been performed to demonstrate its advantages in treating large stones, which is still challenging for manual FURS [30]. However, gender differences in the physiological institutions of the urinary system are not considered in this robot. In addition, it is not sufficiently convenient and intuitive for surgeons to use two separate joysticks to control the 3-DoF motion of the flexible ureteroscope. In the preliminary research, we developed a master-slave robotic system to target FURS and carried out both phantom and animal experiments [31]. This system verified the effectiveness of the robot-assisted FURS. However, the mechanical design is simple and can only provide the basic functions for operations, and the presented control strategy is not sufficient for complex operations.

In this paper, a newly designed master-slave robotic system for FURS is introduced. The requirement analysis is introduced first, based on which the detailed design of the master manipulator and the slave manipulator is illustrated. The following part describes the master-slave mapping and control strategies. The phantom experiments and animal experiments are described and analyzed. The conclusion is drawn in the last section of this paper.

## 2. Materials and Methods

### 2.1. Requirements and Concept Design of the Robotic System

#### 2.1.1. Design Requirement Analysis

**Figure 2:**
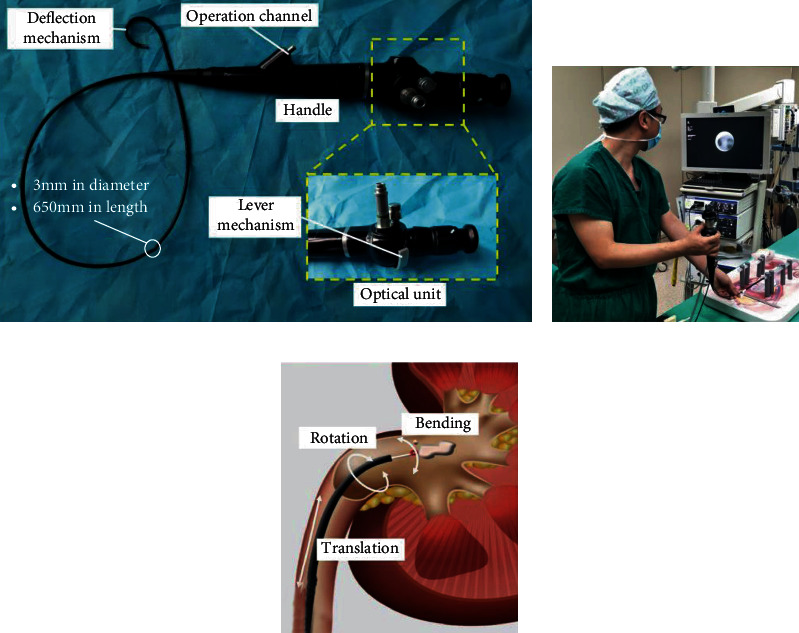
(a) Components of a commercial flexible ureteroscope; (b) the conventional manual FURS in a standing operation mode; (c) the typical three DoFs of the flexible ureteroscope, including translation, rotation, and distal bending.

The slender flexible ureteroscope (typically less than 3 mm in diameter and 650 mm in length) is the essential instrument to examine the internal status of kidneys and treat stones and tumors in FURS procedures. It is typically composed of an embedded optical unit, an operation channel, and a lever mechanism, as shown in Figure 2(a). The lever mechanism is designed to realize the distal bending motion with stainless wires that pass through the ureteroscope.

*(1) Movement Analysis of the Flexible Ureteroscope*. During manual FURS, the surgeon typically holds the control handle with the right hand to carry out in-out motion and overall rotation of the ureteroscope and rotates the lever mechanism to realize the distal bending. The left hand operates the ureteroscope around the urethra orifice to assist the insertion and rotation motion. Based on this analysis, the motion of the flexible ureteroscope can be described by 3 DoFs, which include translation, rotation, and bending, as shown in Figure 2(c). Besides, other instruments, such as fibers, baskets, and guidewires, can be advanced and withdrawn through the working channel inside the ureteroscope to perform stone fragmentation, basket extraction, tissue biopsy, etc.

To investigate the mechanical design requirements, this study summarizes the design criteria on the basis of the manual FURS. The robotic system for FURS is supposed to have a 3-DoF movement to realize the translation, rotation, and bending of the ureteroscope. The motion range for each DoF should reach 200 mm, 360°, and ±270°, respectively, to be able to fully access every part of the upper urinary tract according to the volume of a normal kidney and conventional FURS procedure [32]. In addition, another local translation DoF is necessary for operating auxiliary instruments, whose motion range is about 10 mm according to clinical experience.

*(2) Suitability of Different Ureteroscopes*. A variety of flexible ureteroscopes are available in clinical applications, including reusable and disposable products. The main differences among them refer to the shape and size of the lever mechanism. Therefore, a quick-exchange modular is required for different commercial ureteroscopes.

*(3) Preoperation Adjustment and Safety Considerations*. After the patient is under general anaesthesia and fixed in the operative position, the spatial location of the urinary system is determined. Therefore, the height and inclination angle of the ureteroscope are supposed to be adjustable for various surgical cases, such as with different genders, the height of operating tables, and operative positions.

Meanwhile, in emergency cases, the ureteroscope should be retracted during operation due to safety considerations. The power transmission to rotate the lever mechanism should be cut off in this procedure to free the distal end, and it should be manually operated without any extra power and tools.

#### 2.1.2. Overall Design of the FURS Robot

**Figure 3:**
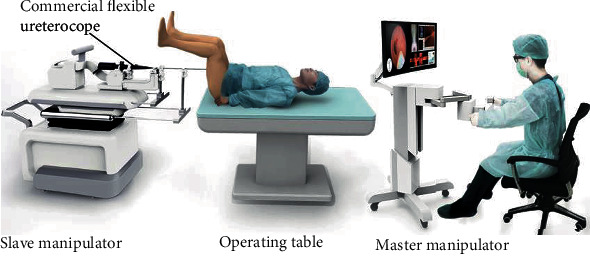
The conceptual design of the robot-assisted system for FURS in a master-slave configuration.

According to the above analysis, an implementation solution of a robotic system is proposed for FURS, as shown in Figure 3. It consists of a master manipulator and a slave manipulator. Commercial flexible ureteroscopes can be mounted on the slave manipulator and maneuvered through the specially designed quick-exchange interface. The surgeon can realize intuitive control through the master manipulator in a sitting position under the navigation of the ureteroscope imaging system.

### 2.2. Design of the Robotic System

#### 2.2.1. Mechanical Design of the Master Manipulator

The master manipulator should offer excellent features in terms of low inertia, counterbalanced gravity, and fine back-drivability. Since the movement of the ureteroscope includes 3 DoFs of translation, rotation, and bending, the master manipulator is also configured with the same arrangement. Considering that the rotational joint usually has excellent back-drivability, finding a translational mechanism driven by a rotational joint becomes the critical issue to develop the master manipulator.

The Sarrus mechanism is a spatial overconstrained mechanism with one pure translational motion capability [33]. All joints in this mechanism are rotational joints. It is a befitting reference for the translation of the master manipulator. To reduce the space occupation, the Sarrus mechanism is redesigned as a planar six-linkage closed-loop mechanism. Under this condition, the number of DoFs of the mechanism can be calculated as

(1) $$\begin{array}{c} F=3\times n-2\times P_{L}=3\times 5-2\times 6=3, \end{array}$$

where *n* denotes the number of moving links and *P*_*L*_ is the number of low-pair linkages.

**Figure 4:**
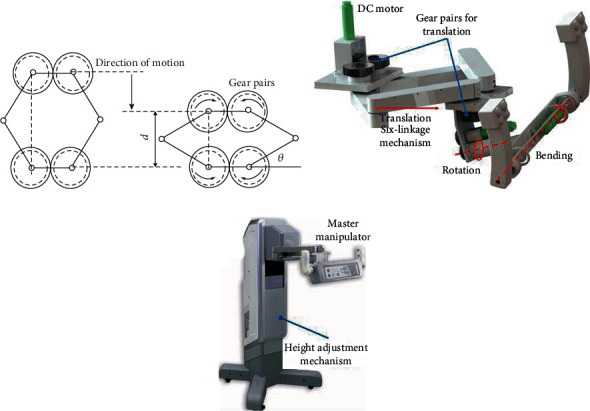
(a) Schematic diagram of the six-linkage mechanism to realize translation by rotation; (b) the design details of the master arm; (c) the mechanical design and configuration of the master.

Therefore, two constraints should be added to the mechanism to limit the movement to a pure translation. Two identical gear pairs were adopted, as shown in Figure 4(a), which can satisfy the above requirements.

To eliminate the influence of gravity on the mechanism and match the translational movement of the flexible ureteroscope, all rotational joints in this mechanism are configured vertically to the ground. Therefore, the configuration of the translation was determined. The relationship between the rotation of the linkage *θ* and the translation *d* can be described as

(2) $$\begin{array}{c} \begin{array}{c} d=2L\sin\theta ,\\ \dot{d}=2\dot{\theta }L\cos\theta , \end{array} \end{array}$$

where *L* represents the length of the 4 identical linkages.

A rotational mechanism was mounted on the end of the above-mentioned six-linkage mechanism to match the rotation motion of the endoscope, as shown in Figure 4(b). The two-handed mode has been adopted to control this mechanism, like driving an automobile. The main advantages of this configuration include the following: (1) the gravity balance has been realized by the symmetric configuration, (2) sufficient control accuracy has been achieved by increasing the distance between the spin axis and the handle, and (3) the stability has been improved by the two-handed mode. Another rotational joint was designed to match the bending motion, as illustrated in Figure 4(b). This configuration can not only improve the stability of bending movement but also avoid the fatigue of the thumb in manual operation.

In addition, the height of the master manipulator on the master console is adjustable to assist surgeons in finding a more comfortable operation pose. The overall configuration of the master console is shown in Figure 4(c).

#### 2.2.2. Development of the Slave Manipulator

Several key issues should be considered to meet the requirements mentioned above in the design of the slave manipulator, including the following: (1) the height and inclination should be adjustable during the preoperation procedure to meet different preoperative requirements, (2) the active mechanism of the robot is supposed to be quickly removed in surgery for evacuation of stone fragments and safety considerations in an emergency, (3) the active mechanism should allow the 3-DoF motion for the endoscope and the operation of auxiliary instruments, and (4) an assistant mechanism is necessary to avoid buckling for inserting the slender endoscope.

**Figure 5:**
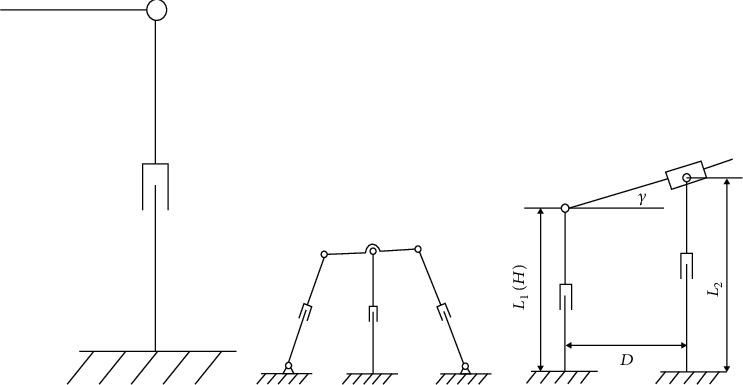
Three 2-DoF mechanisms that can realize adjustment of height and inclination.

*(1) Adjustment of the Height and Inclination*. The slave manipulator should perform a 2-DoF movement for the height and inclination angle adjustment. Some mechanisms can meet this requirement, as illustrated in Figure 5. The first scheme is a serial mechanism with a compact configuration but undesirable stiffness, especially when the translational joint reaches close to the upper limiting position. The second and third schemes both adopt a parallel configuration with a larger bearing capacity, as shown in Figures 5(b) and 5(c). The second scheme occupies a larger space due to the swing of the two branches during adjustment. By contrast, the third scheme eliminates the swing of the branches for less space occupation. Therefore, the third scheme has been adopted in practice. The relationship between the length of the branches and the height and inclination is

(3) $$\begin{array}{c} \begin{array}{c} H=L_{1},\\ \gamma =\arctan\frac{L_{2}-L_{1}}{D}, \end{array} \end{array}$$

where *H* and *γ* represent the height and the inclination angle, respectively. The lengths of the two vertical branches are denoted as *L*_1_ and *L*_2_. The distance between the two branches is *D*.

**Figure 6:**
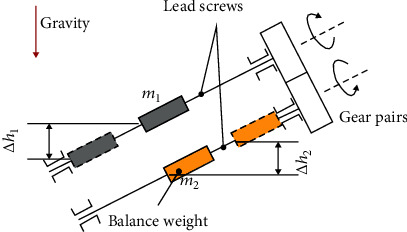
The schematic diagram of the translation joint with gravity balancing mechanism for quick insertion and retraction function.

*(2) Quick Insertion and Retraction Function*. To meet quick insertion and retraction requirements, a passive translational DoF is supposed to be included with a 650 mm motion range. However, the whole passive translational joint tends to slide down by gravity when the inclination happens. To solve this problem, a gravity compensation mechanism has been designed to conserve the gravitational potential energy, as illustrated in Figure 6. During the passive translation procedure, the variation of the gravitational potential energy (GPE) Δ*E*_1_ can be calculated as

(4) $$\begin{array}{c} \Delta E_{1}=m_{1}g\Delta h_{1}, \end{array}$$

where *m*_1_ denotes the mass of the whole part, which is moveable in this procedure. Δ*h*_1_ represents the variation of height.

To compensate for the lost potential energy, the variation of the GPE for the balance weight Δ*E*_2_ is

(5) $$\begin{array}{c} \Delta E_{2}=-\Delta E_{1}=m_{2}g\Delta h_{2}, \end{array}$$

where *m*_2_ and Δ*h*_2_ denote the mass and the height variation of the balanced weight, respectively.

In the actual design, the movements of the two parts are kept equivalent in magnitude but opposite in direction. This constraint condition has been realized by choosing two identical leadscrews with parallel configuration and two identical gears, as shown in Figure 6.

**Figure 7:**
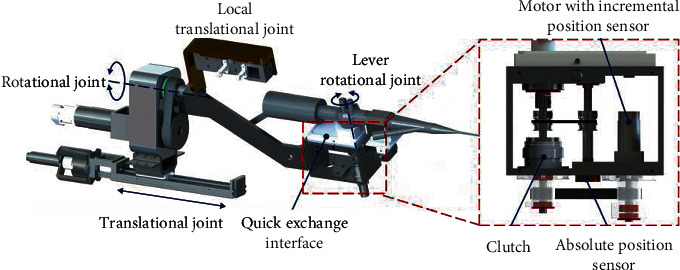
The 3-DoF operational mechanism for ureteroscope.

*(3) Operation of the Flexible Ureteroscope and the Auxiliary Instruments*. The ureteroscope is supposed to be manipulated by the slave manipulator to realize the motions of translation, rotation, and distal bending. A linear module has been utilized to achieve a 220 mm translation motion. A pair of synchronous pulleys have been equipped with the rotational joint for a 450° motion range, which provides more flexibility in surgery. Another rotational joint has been configured for the rotation of the lever mechanism to realize distal bending. A clutch has been configured in this joint for the power interruption requirement. However, the incremental encoder assembled with the servomotor cannot measure the passive motion of the bending joint when the clutch cuts off the power transmission. Therefore, another absolute position sensor has been utilized to measure the rotation of the lever mechanism by cable-driven mechanism, as illustrated in Figure 7. Meanwhile, quick-exchange interfaces have been designed for different ureteroscopes to enhance the adaptability of the robot. During operation, the quick-change interface is bonded to a sterile bag to realize the isolation of the flexible ureteroscope and the slave manipulator to ensure the sterility of the surgical environment. Therefore, only the quick-change interface and the flexible ureteroscope are required to be sterilized instead of the robotic system. The overall scheme and the detailed design for each DoF are shown in Figure 7.

**Figure 8:**
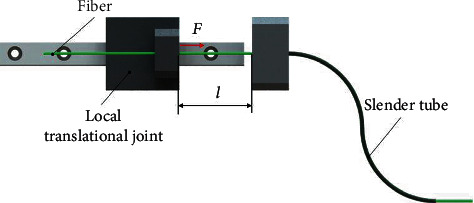
Design of the local translational joint for fiber insertion.

Besides the flexible ureteroscope, there are other required instruments in manual FURS, such as laser fiber for stone fragmentation. To realize the motion of the fiber automatically, a local translational joint has been considered and mounted on the proposed 3-DoF mechanism, which can be directly controlled by the surgeon. A slender tube has been utilized to guide the fiber for insertion from the local joint to the endoscope, as shown in Figure 8. According to Euler's critical load, the longitudinal compression load on the fiber should be less than a critical load to avoid buckling, which can be described as

(6) $$\begin{array}{c} F<F_{\text{cr}}=\frac{\pi^{2}EI}{{\left(\mu l\right)}^{2}}, \end{array}$$

where *F*_cr_, *E*, *I*, *μ*, and *l* denote the critical load, Young's modulus of the fiber, the inertia moment of the cross section of the fiber, the effective length factor, and the length of the unsupported length of the fiber, respectively. In clinical applications, the insertion force is less than 0.5 N [34]. Therefore, the motion range of the local translational joint is determined by the maximum value of *l*. It can be calculated as

(7) $$\begin{array}{c} l\leq \mu \sqrt{\frac{\pi^{2}EI}{F_{\text{cr}}}}=15.55\text{ mm}. \end{array}$$

Therefore, the maximum movement distance of the local translational joint is determined as 15 mm to avoid buckling. The values adopted in Equation (7) are listed in Table 1.

**Figure 9:**
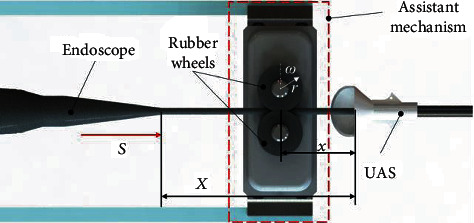
Design of the assistance mechanism for the insertion of the ureteroscope.

*(4) Design of the Assistance Mechanism for Insertion*. Similar to the situation in fiber insertion, the ureteroscope may also experience buckling during the insertion procedure for the same reason. Therefore, an assistance mechanism has been designed beside the ureteral access sheath (UAS) to assist the insertion, like a hand around the urethra orifice, as shown in Figure 9. Two rubber wheels are utilized, one of which is driven by a servomotor, to insert and pull out the ureteroscope through friction, which is adjustable by changing the distance between the two wheels. In this configuration, the length of the slender part is shortened from a variable *X* to a smaller constant *x* to avoid bucking.

**Figure 10:**
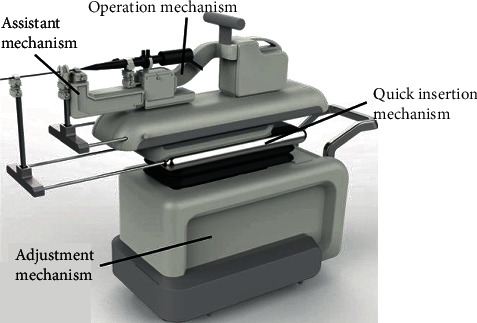
The integration of the slave manipulator.

Based on the above description, the slave manipulator has been integrated, as shown in Figure 10. The design parameters of the master and slave manipulators are listed in Table 2. The motor and electrical component selections have been listed in Table 3.

#### 2.2.3. Master-Slave Mapping and Control Strategies

**Figure 11:**
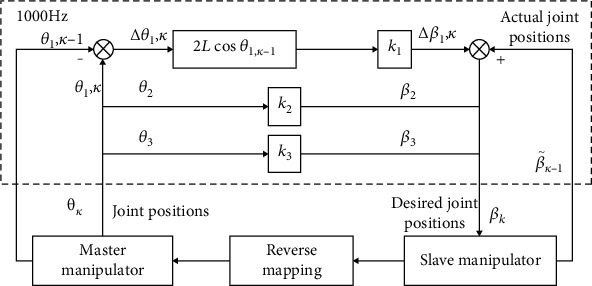
The applied joint-to-joint control strategy of the robotic system.

*(1) Master-Slave Mapping Strategy*. A proper mapping strategy can make the operation more manageable and more precise. Therefore, a joint-to-joint mapping strategy has been established for this robot to guarantee the intuitive control of the general FURS operation. The overall master-slave mapping strategy is illustrated in Figure 11. The details are presented as follows.

For the translation motion, an incremental-proportional strategy has been designed. The proportional strategy can convert a large-scaled movement at the master manipulator to a small-scaled movement at the slave side. This can improve the operation accuracy, which is essential when the surgeon is carrying out a delicate operation. The incremental part is used to reposition the master manipulator. This function is essential when the master manipulator encounters the motion limit or the surgeon is in an uncomfortable pose. The mapping relationship can be described as

(8) $$\begin{array}{c} \Delta \beta_{1,k}=k_{1}\cdot 2L\cos\theta_{1,k-1}\Delta \theta_{1,k}, \end{array}$$

where Δ*β*_1,*k*_ and Δ*θ*_1,*k*_ denote the motion increments for the slave and master manipulators, respectively. The position of the translational joint of the master manipulator before repositioning is denoted as *θ*_1,*k*−1_. The parameter *k*_1_ represents the proportional parameter.

For the rotational and bending motions, a proportional strategy is applied to match the different motion ranges between the two sides. Hence,

(9) $$\begin{array}{c} \left[\begin{array}{c} \beta_{2}\\ \beta_{3} \end{array}\right]=\left[\begin{array}{cc} k_{2} & 0\\ 0 & k_{3} \end{array}\right]\cdot \left[\begin{array}{c} \theta_{2}\\ \theta_{3} \end{array}\right], \end{array}$$

where *β*_2_ and *β*_3_ denote the positions of rotational and bending joints of the slave manipulator, respectively. Positions of the corresponding joints of the master manipulator are denoted as *θ*_2_ and *θ*_3_. The proportional parameters are *k*_2_ and *k*_3_, respectively.

However, the mapping between rotation and bending may be interrupted due to manual intervention, such as the quick retraction procedure. To maintain the mapping consistency before and after the intervention, a reverse mapping strategy is built in the manual intervention procedure. It can be described as

(10) $$\begin{array}{c} \left[\begin{array}{c} \theta_{2}\\ \theta_{3} \end{array}\right]=\left[\begin{array}{cc} k_{2}^{-1} & 0\\ 0 & k_{3}^{-1} \end{array}\right]\cdot \left[\begin{array}{c} \beta_{2}\\ \beta_{3} \end{array}\right]. \end{array}$$

*(2) Control Strategy for the Bending Joint of the Slave Manipulator*. During the manual evacuation procedure, the power transmission of the bending joint is cut off by the clutch mechanism. Under this condition, the bending joint can move freely, and the endoscope can be withdrawn safely. The actual position of this joint, meanwhile, can be recorded by the absolute sensor. Once the transmission is regained, the incremental encoder value is set to be the same as that of the absolute sensor for accurate position control. Therefore, the actual position of the bending joint can be calculated as

(11) $$\begin{array}{c} \begin{array}{c} \beta_{3,k}=\left(1-\lambda \right)\delta_{3,k-1}+{\lambda \varphi }_{3,k-1},\\ \delta_{3,k}=\beta_{3,k}, \end{array} \end{array}$$

where *δ*_3,*k*−1_ and *φ*_3,*k*−1_ denote the actual joint positions of the incremental and absolute sensors at time *k* − 1, respectively. The actual joint position of the bending joint at time *k* is denoted as *β*_3,*k*_. The clutch status is represented by *λ*, which is set as 1 when the power transmission is interrupted.

*(3) Kinematic Relation between the Translational Joint and the Assistance Mechanism*. As mentioned above, an assistant mechanism with two rubber wheels has been designed for the insertion procedure. The relationship between the translation distance of the translational joint *S* and the rotation angle of the wheels *ω* is

(12) $$\begin{array}{c} S=\omega r, \end{array}$$

where the radii of the two rubber wheels are both *r*.

However, the endoscope may be stretched and damaged since the motions are not strictly synchronous in real time. Therefore, a constant distance has been introduced to loosen the ureteroscope. In the insertion procedure, the translational distance *S* generated by the translation joint is longer than that of the rotation wheels, thus avoiding the tension trend and protecting the endoscope. However, the condition should be the opposite in the withdrawing procedure. Therefore, the actual rotation angle of the wheel can be described as

(13) $$\begin{array}{c} \tilde{\omega }=\frac{S-\mathrm{sgn}\left(\dot{S}\right)\Delta }{r}, \end{array}$$

where Δ is a generally positive constant displacement and $\tilde{\omega }$ denotes the actual rotation angle. The velocity of the insertion is denoted as $\dot{S}$.

### 2.3. Experiment Design

**Figure 12:**
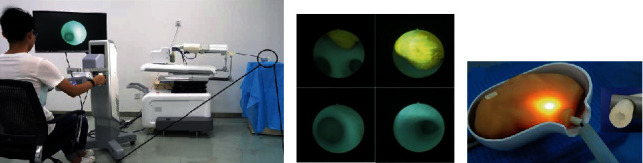
Setup of the phantom experiment: (a) robotic operation of one volunteer; (b) field of view of the flexible ureteroscope; (c) the phantom utilized in the experiment.

Two experiments have been designed to investigate the usability and operability of clinical applications of the proposed robotic system. The experiment setup is shown in Figure 12.

The first experiment was set to research the usability of the designed system towards inexperienced operators in contrast to manual operations. Twenty engineering undergraduates with no prior knowledge about FURS were invited and randomly divided into two groups. The first group was set as the control group to finish the FURS procedure manually. The second group was arranged to complete this procedure through the designed robotic system. A renal phantom with fabricated stones by 3D printing technology based on a patient's CT data was utilized in this experiment to simulate the actual upper urinary tract. A flexible ureteroscope (URF-P5, Olympus, Japan) was employed to perform FURS by manual and robotic operations, respectively. Before the experiments, all volunteers were introduced to basic knowledge of the renal collection system. The second group was taught about the usage of the robotic system. For both groups, the renal examination began at the ureter, and the volunteers controlled the ureteroscope to reach the pelvis to screen each calix and locate the kidney stones. After all kidney calices were examined, the ureteroscope was retracted and the experiments stopped. Each volunteer repeated the examination in 6 cases with a 1 min interval. The procedure time for each examination was recorded and analyzed.

**Figure 13:**
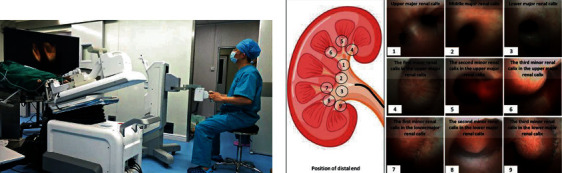
(a) Setup of the phantom experiment; (b) field of view from the flexible ureteroscope.

The second experiment was arranged to further evaluate the operability of the proposed system. An experienced surgeon, who performed more than 5000 clinical FURS procedures, was invited to join this experiment. Two healthy pigs (42 kg and 28 kg in weight) were used in this experiment to provide human-like renal collection systems. The surgeon was trained to gain the operation skills of the robotic system before the experiment began. During the experiment, the pigs were anesthetized and then immobilized on the operating table. A guidewire was inserted into the ureter close to the pelvis, and a ureteral access sheath (12/14 F, Cook, USA) and a flexible digital ureteroscope (Pusen, Zhuhai, China) were advanced to follow the guidewire and access the kidney in succession. Then, the slave manipulator was moved beside the operating table and adjusted to aim at the urethra. The ureteroscope was then mounted on the slave manipulator, and the preoperative work was ready. After that, the surgeon was invited to control the robotic system to perform FURS. All kidney calices needed to be reached in a single diagnosis, and this procedure was repeated twice for each kidney of the pigs. Therefore, 8 trials have been performed in total. The duration from inserting the guidewire to mounting the ureteroscope on the slave manipulator was recorded and defined as the preoperative time. The duration of the examination procedure was also recorded and named as the operative time.  Figure 13 illustrates the animal experiment setup.

## 3. Results and Discussion

### 3.1. Results

**Figure 14:**
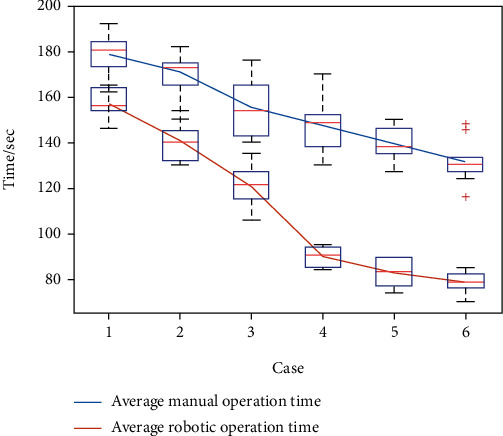
Time comparison between the manual and robotic operations in the phantom experiment.

The average time, the median time, and standard deviations of both groups were calculated for each case during the first experiment, as listed and illustrated in Table 4 and Figure 14, respectively. For the manual group, the average duration of 10 volunteers was decreased from 178.4 s (in the first case) to 131.4 s (in the sixth case). By contrast, the average duration for the second group decreases from 156.8 s to 78.7 s. Thus, each average duration in the second group is less than its corresponding value in the first group. In addition, there is an apparent decline between the third case and the fourth in the second group.

**Figure 15:**
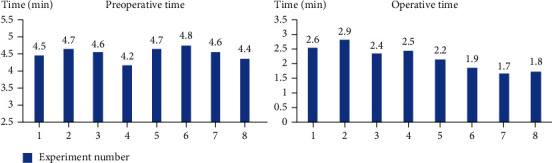
The preoperative time and operative time values in the animal experiment.

Eight experimental trials on animals were attempted, and all kidney calices were successfully localized and observed. The average preoperative time is 4.56 min within the range of 4.2 to 4.8 min. The average operative time is 2.25 min within the range of 1.8 min to 2.9 min, as shown in Figure 15. Both pigs were still alive after the surgical procedure, and no further complications occurred in either of them.

### 3.2. Discussion

FURS is a challenging NOTES procedure with complex operations and poor ergonomics. Surgeons should be intensively trained to master this technique. A robotic system has been elaborated and examined to improve the ergonomics and make it easier for inexperienced operators.

For the phantom experiment, there is an apparent difference between the manual and robotic operations. In general, the average time in the robotic group is less than that in the manual group, which may be ascribed to the intuitive robotic operation mode. Meanwhile, the obvious decline in the robotic group indicates that inexperienced operators can master such skills in a shorter duration, confirming that the DoF configuration of the master manipulator is more acceptable and can shorten the learning curve.

The average preoperative time reflects the practical function of the height and inclination adjustment. In the preoperative work, the slave manipulator needed to be adjusted to aim at the urethra. All preoperative times are relatively stable, which reasonably reflects the acceptable performance of the adjustment function. The operative time in the animal experiment is a little longer than that of the phantom experiment due to the influence of the unclear field of view and bubbles caused by saline irrigation and bleeding in the practical operations. It is also worth noting that the operative time is gradually shortened with the increase of operating trials and skills. In addition, the comfort level of the legs and waist has been significantly improved according to the surgeon's feedback due to the sitting position and ergonomic design of the master arm.

There are also some limitations in this research. While the master manipulator facilitates the operation, further research will be developed to compare the effectiveness of the special master manipulator with that of commercial master devices. Different mapping strategies will also be developed and compared to explore better intuitiveness. For the comfort level evaluation of the robotic system, more quantitative analysis will be carried out, such as fatigue tests based on electromyography sensors and questionnaire surveys for surgeons about their feelings and advice.

## 4. Conclusion

A novel robot-assisted surgical system has been proposed and implemented with a master-slave configuration to target the FURS procedures. The advantages of the designed system enable it to provide excellent adaptability and convenient adjustment to cope with different ureteroscopes and fit various patients and cases. Both phantom and animal experiments have been conducted to demonstrate its advantages of an easy-to-use manner, shortened learning curve, and enhanced comfort for the legs and waist of surgeons, demonstrating its potentials for clinical applications. Future work also involves applying fiber Bragg grating-based force- and shape-sensing techniques to enable force feedback and closed-loop control for accurate operations [35, 36], as well as 5G-enabled teleoperation [37].

## Figures and Tables

**Table 1:** Values of parameters and variables in Equation (7).

*E*	*I*	*μ*	*F*_cr_
4.0 GPa	3.07 × 10^−3^ mm^4^	1.0	0.5 N

**Table 2:** Design parameters of the proposed robotic system.

Motion ranges for master manipulator		Motion ranges for slave manipulator	
Translation	173.2 mm	Translation	220 mm
Rotation	120°	Rotation	450°
Bending	90°	Bending	90°
/	/	Height adjustment	574-925 mm
/	/	Inclination adjustment	0-45°
/	/	Translation adjustment	650 mm

**Table 3:** Motor and electrical component selections of the proposed robotic system.

Joint/electrical components	Motor/electrical component selection
Translation joint in the master manipulator	Maxon DCX22S, with a gearbox ratio of 21 : 1
Rotation joint in the master manipulator	Maxon DCX22S, with a gearbox ratio of 21 : 1
Deflection joint in the master manipulator	Maxon DCX22S, with a gearbox ratio of 21 : 1
Height adjustment in the master manipulator	Maxon RE35
Translation joint in the slave manipulator	Maxon DCX22S, with a gearbox ratio of 21 : 1
Rotation joint in the slave manipulator	Maxon DCX22S, with a gearbox ratio of 231 : 1
Deflection joint in the slave manipulator	Maxon RE-max17, with a gearbox ratio of 100 : 1
Height and inclination adjustment in the slave manipulator	Maxon RE35
Insertion assistance mechanism in the slave manipulator	Maxon RE-max17, with a gearbox ratio of 100 : 1
Amplifiers in both master and slave manipulators	ACK-055-06, Copley Controls
Motion controller	Power Clipper, Delta Tau

**Table 4:** Results of the phantom experiment (unit: sec).

No.	Manual operation		Robotic operation	
	Average	Median ± standard variations	Average	Median ± standard variations
1	178.4	180.5 ± 8.5	156.8	156.0 ± 6.2
2	170.6	172.5 ± 9.1	140.7	140.0 ± 8.0
3	155.2	154.0 ± 11.6	120.6	121.5 ± 8.9
4	147.2	148.5 ± 11.4	89.9	90.5 ± 4.0
5	139.6	138.0 ± 7.2	82.7	83.5 ± 5.6
6	131.4	130.0 ± 9.0	78.7	78.5 ± 4.5

## Data Availability

The data used to support the findings of this study may be released upon application to the first author, Jianchang Zhao, who can be contacted at zhaojianchang@tju.edu.cn.
